# Quality of Life of Patients Undergoing Heart Valve Interventions: An Integrative Review of Studies Using the 36-Item Short Form Health Survey (SF-36) Questionnaire

**DOI:** 10.7759/cureus.97161

**Published:** 2025-11-18

**Authors:** Andra D Marinescu, Victor S Costache

**Affiliations:** 1 Faculty of Medicine, Doctoral School of Medicine, Titu Maiorescu University, Bucharest, ROU; 2 Department of Pneumology, Pneumology Hospital Sibiu, Sibiu, ROU; 3 Faculty of Medicine, Department of Cardiovascular Surgery, Titu Maiorescu University, Bucharest, ROU; 4 Department of Cardiovascular Surgery, Sanador Clinical Hospital, Bucharest, ROU

**Keywords:** cardiac interventions, health-related quality of life (hrqol), heart valve surgery, hrqol sf-36, integrative review, minimally invasive surgery, patient-reported outcomes (pros), quality of life, sf-36 questionnaire, valvular heart disease

## Abstract

Valvular heart disease represents a significant burden on global health, and its impact on health-related quality of life (HRQOL) is increasingly recognized in clinical decision-making. This integrative review synthesized outcomes measured with the 36-Item Short Form Health Survey (SF-36). A structured PubMed search was conducted in November-December 2024 and updated in September 2025; studies published from 2014 onward that reported SF-36 after heart valve interventions were included. Twelve studies met the criteria, spanning surgical and transcatheter approaches and both mechanical and biological prostheses.

Across studies, early (≤1 month) improvements were evident, with the largest gains typically in physical domains. Mid-term (six to 12 months) improvements were generally sustained, and differences between transcatheter and surgical approaches tended to attenuate, with no consistent between-group differences by six to 12 months in studies reporting adjusted comparisons. Age-related patterns were observed: older patients started lower and showed smaller absolute gains in physical domains, while mental health changes were more modest and often comparable across ages. Findings comparing mechanical versus biological valves were heterogeneous and likely influenced by confounding (age, comorbidity, anticoagulation). Longer-term (>12 months) trajectories remain uncertain due to limited longitudinal data.

Limitations include the single-database search restricted to English-language and free full-text articles, variability in follow-up windows and reporting, and the absence of a formal meta-analysis or risk-of-bias grading. Despite these constraints, current evidence indicates that heart valve interventions improve HRQOL, supporting shared decision-making and the need for standardized time points, adjusted analyses, and longer follow-up to refine comparative inferences.

## Introduction and background

Valvular heart disease imposes a significant burden on global health, affecting over 60 million individuals worldwide [1]. Among the myriad of challenges associated with valvular disorders, the impact on health-related quality of life stands as an important aspect warranting thorough investigation [2]. Quality of life encompasses multifaceted dimensions of physical, mental, and social well-being, providing valuable insights into patients' subjective experiences and functional outcomes [3]. In the context of valve disease, understanding the nuances of quality of life becomes paramount, as it influences treatment decisions, expected patient outcomes, and overall patient-centered care [4].

The management of valve disease often involves a spectrum of interventions, ranging from pharmacotherapy to surgical or transcatheter procedures, aimed at alleviating symptoms, preserving cardiac function, and improving quality of life [5]. While advancements in surgical techniques and transcatheter interventions have revolutionized the treatment landscape for valve disease, the impact of these interventions on patients' quality of life remains a subject of active investigation [6]. Studies exploring quality of life outcomes following valve surgery have provided valuable insights into the efficacy and patient-reported benefits of different interventions, shedding light on the complex interplay between clinical outcomes and subjective well-being [7,8].

Despite the growing body of literature addressing quality of life in the context of valve disease and surgery, several knowledge gaps persist [9]. The variability in study methodologies, patient populations, and measured outcomes hinders the comprehensive understanding of quality-of-life outcomes. Additionally, the dynamic nature of quality-of-life assessment, encompassing preoperative, perioperative, and postoperative phases, requires longitudinal studies using standardized instruments in order to capture the trajectory of patients' well-being over time. The 36-Item Short Form Health Survey (SF-36) is a validated, generic health-related quality-of-life instrument developed within the Medical Outcomes Study and widely used across cardiovascular cohorts. It comprises eight domains, physical functioning, role physical, bodily pain, general health, vitality, social functioning, role emotional, and mental health, that can also be summarized as physical and mental component scores. Its widespread adoption enables comparison across interventions and time points, while scoring conventions and version updates should be considered when interpreting results [3,10].

This integrative review synthesized postoperative outcomes measured with the SF-36 questionnaire among patients undergoing heart valve replacement or repair. A structured Medical Literature Analysis and Retrieval System Online (MEDLINE) and Excerpta Medica database (Embase) search was conducted between November and December 2024 and updated during September and November 2025, and identified studies published from 2014 onward that reported SF-36 results after surgical (sternotomy, mini-thoracotomy, fully endoscopic) or transcatheter interventions. Twelve studies involving approximately 1,300 adult patients met the inclusion criteria. Most were prospective observational cohorts with heterogeneous follow-up intervals and analytic methods; therefore, data were narratively synthesized, and no formal risk-of-bias tool or meta-analysis was applied.

Across studies, early (≤1 month) postoperative improvements were consistently reported, with the largest gains typically observed in the physical functioning and role-physical domains (mean increases of 10-25 points on a 0-100 scale). Mid-term improvements (six to 12 months) were generally sustained, and differences between transcatheter and surgical approaches tended to attenuate, with no consistent between-group differences by six to 12 months in studies reporting adjusted comparisons. Mental health-related domains showed smaller but steady gains over time. Comparisons between mechanical and biological valves yielded heterogeneous results, likely confounded by age, comorbidity, and anticoagulation status. Longer-term trajectories (>12 months) remain insufficiently characterized due to limited longitudinal data.

The synthesis was limited to free full-text articles in English, variability in reporting formats and follow-up timing, and the lack of publication bias assessment or formal quality grading.

Despite these constraints, current evidence indicates that heart valve interventions are associated with clinically meaningful improvements in health-related quality of life (HRQOL), especially in physical domains. Standardized follow-up intervals, adjustment for confounding, inclusion of diverse patient profiles, and longer-term evaluations are needed to refine comparative inferences and guide patient-centered decision-making in valvular heart disease.

## Review

Materials and methods

As part of this study, an integrative literature review was conducted, consisting of three phases that were structured to address the research question regarding the HRQOL post-surgery for patients undergoing different types of cardiac valve interventions.

In formulating the guiding question, the study employed the Population, Intervention, Control, and Outcomes (PICO) strategy [11]. The focus of the guiding question was to “determine to which capacity heart valve surgery affected the quality of life of patients” undergoing this type of intervention.

A thorough search was performed in the Medical Literature Analysis and Retrieval System Online (MEDLINE; via PubMed) from November to December 2024. To ensure accessibility and transparency, inclusion criteria were restricted to articles published in English and available as free full texts, although this may introduce selection bias and limit comprehensiveness, as relevant studies indexed in other databases (e.g., the Excerpta Medica database (Embase), Scopus, and Web of Science) or published in other languages may not have been captured. This is addressed further in the Limitations section. The integrative search utilizes both controlled and uncontrolled descriptors in conjunction with Boolean operators. The used keywords were (“quality of life”) OR (“health-related quality of life”) OR (“HRQOL”) AND (“heart valve prosthesis implantation”) OR (“heart valve prosthesis”) OR (“heart valve surgery”) AND (“SF-36”) OR (“SF-36 questionnaire”) OR (“SF 36”) OR (“SF 36 form”). This search followed a structured approach involving three phases, as mentioned before. The search was updated on 12^th^ September, 2025, and the database was expanded to include Embase, using the same string limited to the last 12 months, to incorporate any newly published studies. The full search strategy, with exact strings (including synonyms such as transcatheter aortic valve replacement (TAVR) or transcatheter aortic valve implantation (TAVI), mitral valve replacement (MVR) or transcatheter mitral valve replacement (TMVR), transcatheter edge-to-edge repair system for mitral regurgitation (MitraClip™), transcatheter edge-to-edge repair system for tricuspid regurgitation (TriClip™), filters, and run dates, is provided in Table 1.

**Table 1:** Database search strategy Three-block strategy: (quality-of-life terms) AND (valve intervention terms) AND (SF-36 terms). Exact Boolean strings and run dates are shown, and the syntax reflects the exact PubMed query used, including Medical Subject Headings (MeSH) and title/abstract (tiab) terms. MEDLINE: Medical Literature Analysis and Retrieval System Online; NCBI: National Center for Biotechnology Information

Database / Interface	Coverage	Full Boolean query	Filters	Run date	Notes
PubMed (MEDLINE) / NCBI	2014-2024	(three-block: QoL AND valve AND SF-36) ("Quality of Life"[MeSH] OR "quality of life"[tiab] OR "health-related quality of life"[tiab] OR HRQOL[tiab]) AND ("Heart Valve Prosthesis Implantation"[MeSH] OR "heart valve prosthesis implantation"[tiab] OR "heart valve prosthesis"[tiab] OR "heart valve surgery"[tiab] OR "aortic valve replacement"[tiab] OR SAVR[tiab] OR TAVR[tiab] OR TAVI[tiab] OR "mitral valve repair"[tiab] OR "mitral valve replacement"[tiab] OR MVR[tiab] OR TMVR[tiab] OR MitraClip[tiab] OR TriClip[tiab] OR "tricuspid valve repair"[tiab] OR TTVR[tiab]) AND ("SF-36"[tiab] OR "Short Form 36"[tiab] OR "SF-36 questionnaire"[tiab] OR "SF 36"[tiab] OR "SF 36 form"[tiab])\AND "Humans"[MeSH]	Language: English; Results by year: last 10 years - 2014–(auto-window to 2024-11-28); Text availability: Free full text	28-Nov-24	Records screened per eligibility criteria.
PubMed (MEDLINE) / NCBI	Last 12 months (auto-window to 2025-09-12)	Same Boolean string as above	Language: English; Results by year: Last 12 months; Text availability: Free full text	12-Sep-25	One new eligible study identified and added to review.

These phases encompassed delineating the research question, establishing clear inclusion and exclusion criteria, systematically scouring the chosen database for relevant studies, defining criteria for information extraction based on the research query, meticulously selecting and evaluating studies, interpreting the findings, and synthesizing the accumulated knowledge.

Articles deemed eligible for inclusion were original research manuscripts focusing on the assessment of health-related quality of life among adult patients undergoing valve surgery using the SF-36 questionnaire [10]. The pathology for which patients underwent valve surgery included in the study was degenerative valve disease or rheumatic valve disease. The study specifically targeted articles published from 2014 onwards and accessible in English with full-text availability at no cost. Only studies that reported outcomes using the SF-36 questionnaire were included. Some studies also administered additional instruments (e.g., EuroQol 5-Dimension Questionnaire (EQ-5D), Kansas City Cardiomyopathy Questionnaire (KCCQ), Cardiac Anxiety Questionnaire (CAQ)), but only SF-36 data were extracted and synthesized.

The SF-36 was selected as the focus of this review due to its extensive use in cardiovascular and surgical cohorts, its inclusion in multiple valve intervention studies, and its established interpretive thresholds (e.g., ≥10-point change representing clinically meaningful improvement in physical domains) [10].

Previous reviews have addressed HRQOL in valvular disease broadly or focused on specific procedures (e.g., TAVI vs. surgical aortic valve replacement (SAVR)), but very few have exclusively synthesized SF-36 outcomes across both surgical and transcatheter valve interventions, valve types, and age groups [8,9]. To fill this gap, the present review summarizes domain-level SF-36 changes after heart valve surgery and transcatheter therapies, identifies trends across different patient subgroups and techniques, and highlights unresolved methodological and clinical questions for future research.

No transformations were applied; results are presented as reported in the original studies. This stringent criterion ensured a contemporary and accessible selection of literature for review. Initial screening was based on titles containing pertinent descriptors, followed by abstract review. Full-text articles meeting the criteria and addressing HRQOL following valve surgery, employing validated assessment tools, were subjected to detailed scrutiny. The search strategy did not include manual searches of journals or adding further references beyond the initially identified studies.

The exclusion criteria for screened articles included studies involving other species, pediatric populations, systematic reviews, meta-analyses, comments, and letters to the editor. Also, in terms of methodology, articles employing qualitative approaches or using only other types of questionnaires were excluded.

**Figure 1:**
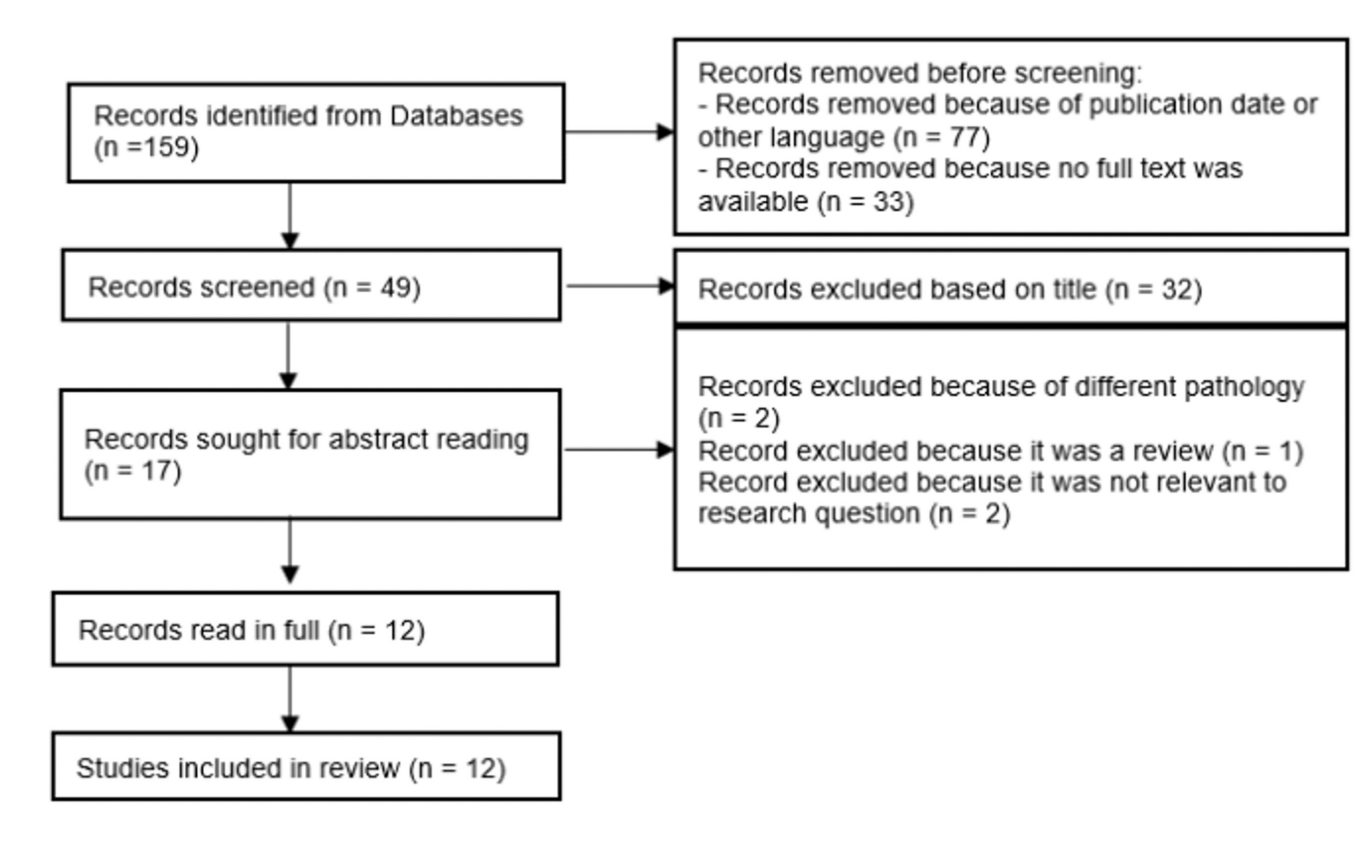
Adapted PRISMA flowchart used for selecting the studies from 2014–2024 PRISMA: Preferred Reporting Items for Systematic Review and Meta-Analyses Records excluded because of different pathology (n = 2) [13,14]. Record excluded because it was a review (n = 1)[15]. Record excluded because it was not relevant to research question (n = 2) [16,17].

The study meticulously adhered to the guidelines outlined in the Preferred Reporting Items for Systematic Review and Meta-Analyses (PRISMA) [12] flowchart to transparently delineate the search process, as can be seen in Figure 1. 

Results

Data extraction was carried out manually to enable descriptive analysis of variables related to study characteristics and evaluation of health-related quality of life. Data extraction followed a structured approach based on authors, study delineation, patient count, correction type, methodology, and outcomes. Each study's results were briefly summarized alongside these parameters. Only SF-36 outcomes were extracted and synthesized. Results from other instruments (e.g., EQ-5D, KCCQ, CAQ) are described narratively when relevant but were not tabulated or pooled. The primary data extracted from the selected articles are summarized in Table 2, adhering to the specified inclusion criteria.

**Table 2:** Synthesis of the studies included in the review according to authors, delineation, patients, and results. AS: aortic stenosis; GK: Star GK (mechanical valve type); KCCQ: Kansas City Cardiomyopathy Questionnaire; LVEF: left ventricular ejection fraction; PARTNER 3: Placement of Aortic Transcatheter Valves 3 (randomized clinical trial); PF: physical functioning (subscale of SF-36); RCT: randomized controlled trial; RP: role-physical (subscale of SF-36); SF-36: 36-Item Short Form Health Survey (SF-36); SJM: St. Jude Medical (mechanical valve type); TTVR: transcatheter tricuspid valve repair The 2025 systematic search update identified one additional eligible study, which was incorporated into the qualitative synthesis [29].

Authors	Delineation	Patients	Results
Akowuah et al. [18]	RCT in 10 tertiary care institutions in the UK carried out between November 2016 and January 2021	330 patients (166: mini-thoracotomy, 164: sternotomy) with data available in full for 278 respondents	Improvement in T scores was observed in both groups throughout the entire timeline up to the one-year follow-up. At the one-year mark, both groups exhibited similar mean changes from baseline in T scores, indicating similar efficacy of the interventions employed.
Purkayastha et al.[19]	Prospective, observational study carried out between 2013 and May 2019 in India	47 patients with data available in full for 35 respondents	Mean scores for mental and physical domains indicate good mental and physical health among respondents at a mean follow-up of 3 ± 1.7 years.
Kitamura et al.[20]	A study carried out at the Heart Center Leipzig at the University of Leipzig, between June 2016 and January 2020	125 patients with full data available for 115 respondents	All scores improved, with subcategories physical role and general health showing the strongest favorable response, indicating that TTVR effectively addresses physical limitations and overall well-being in patients.
Huang et al.[21]	A retrospective study carried out between January 2013 to December 2015 in China	172 adult patients (SJM valve for 87 patients and Star GK valve for 85 patients)	Scores at the one-year follow-up indicate good mental and physical health among respondents, with no significant differences between the two groups, indicating the effectiveness of both types of mechanical mitral valves.
Wang et al.[22]	A retrospective study carried out in a single center in China between June 2017 to February 2018	78 patients aged 60 to 70 years (40 patients within the mechanical valve group and 38 patients within the biological valve group)	At discharge, both groups had similar scores. However, after one year, the biological valve group showed significantly higher SF-36 scores, indicating better long-term quality of life for this group. Conversely, the mechanical valve group exhibited significantly higher levels of cardiac anxiety compared to the biological valve group.
Bento et al.[23]	A single‐center retrospective study of octogenarians carried out between January 2011 and December 2015 in Hospital de Santa Marta, do Centro Hospitalar de Lisboa Central	163 consecutive octogenarian patients with full data available for 81 respondents	All dimensions of SF-36 showed statistically significant improvement throughout follow-up when compared to the preoperative period. The mental component exhibited statistically significant improvement at six- and twelve-month follow-ups compared to the preoperative period, indicating a reduction in mental burden associated with the disease following surgery.
Huang et al.[24]	A single-center retrospective cohort study of patients undergoing mitral valve surgery was carried out between January 2019 and December 2019 in China	163 patients (78 patients - total endoscopic approach, 85 patients - median sternotomy)	Within the two groups, significant differences were observed in the scores for bodily pain and mental health subscales, denoting reduced postoperative bodily discomfort and enhanced mental well-being consequent to the modified total endoscopic approach.
Korteland et al.[25]	A single-center cohort study in the Netherlands cross-sectionally surveyed one to 10 years after aortic valve surgery carried out between January 2001 and December 2011	457 patients with full data available for 240 respondents (190 patients within the mechanical valve group and 50 patients within the biological valve group)	Patients who underwent aortic valve replacement displayed differing health-related quality of life outcomes compared to the general Dutch population, with lower scores in physical health but higher scores in mental health. Furthermore, the association between increased time post-surgery and improved mental health suggests the potential for long-term adaptation and positive outcomes in these patients.
Blokzijl et al.[26]	An observational, multicenter, cohort study carried out in the Netherlands between January 2011 and January 2015	889 patients were classified into 3 age groups: <65 (232 patients), 65-79 (554 patients), and ≥80 years (113 patients).	The study reveals notable improvements in both physical and mental health scores following surgery across all patient groups. However, at the one-year follow-up, a significant portion of patients, particularly in older age groups, showed smaller absolute gains and remained at lower absolute physical scores at one year. indicating the influence of age-related factors on long-term outcomes.
Baron et al. [27]	The PARTNER 3 trial was carried out between March 2016 and October 2017 and was a multicenter randomized trial.	1000 patients with data available in full for 943 respondents (494 patients in the transcatheter group and 449 in the surgical group).	At 1-month post-procedure, transcatheter patients demonstrate significant improvements in physical and mental health compared to surgical patients. However, by six and 12 months, no discernible differences are observed between the two groups, suggesting comparable long-term health outcomes between the two procedures.
Abd Al Jawad et al.[28]	A prospective nonrandomized study was carried out between May 2014 and December 2020 in Egypt.	260 patients with full data available for 189 respondents (96 patients in the mini-thoracotomy group and 93 patients in the mini-sternotomy group)	The study demonstrates significant health improvement in the mini-thoracotomy group compared to baseline measurements, with notably better physical functioning than the mini-sternotomy group. These findings suggest that mini-thoracotomy may offer superior outcomes in terms of physical health, emphasizing its potential as a favorable surgical approach for selected patients.
Sævik et al.[29]	Prospective observational study, Oslo University Hospital, 2017–2020	88 elderly patients (mean age 80 ± 6) with severe symptomatic AS, preserved LVEF	About half of the patients achieved clinically meaningful improvement in PF (50%) and RP (52%). Patients with lower baseline PF/RP scores had the greatest improvement. No significant associations with age, comorbidities, or risk scores.

Detailed domain-level SF-36 results are provided in Table 3.

**Table 3:** Detailed SF-36 domain-level results across included studies AVR: aortic valve replacement; BL: baseline (pre-intervention); CAQ: Cardiac Anxiety Questionnaire; CI: confidence interval; Δ: change or difference (delta); EA: endoscopic approach; EQ-5D: EuroQol 5-Dimensions questionnaire; FU: follow-up; GH: general health; KCCQ: Kansas City Cardiomyopathy Questionnaire; MCS: mental component summary; MHC: mental health component; MLHFQ: Minnesota Living with Heart Failure Questionnaire; NL: the Netherlands; NRS: Numeric Rating Scale; PCS: physical component summary; PHC: physical health component; QOL: quality of life; RAND: research and development; SA: sternotomy approach; SAVR: surgical aortic valve replacement; SCAR: Scar Cosmesis Assessment and Rating scale; SF-36: 36-Item Short Form Health Survey (SF-36); SJM: St. Jude Medical (mechanical valve type); TAVI: transcatheter aortic valve implantation; TTVR: transcatheter tricuspid valve repair/replacement; For interpretability, SF-36 domain scores are presented on their native 0–100 scale when available; studies reporting norm-based T-scores are labeled accordingly, and no cross-metric transformations were applied.

Author, Year of publication	Intervention/N/Follow-up	Other QOL questionnaires used	Physical Functioning (PF)	Role Physical (RP)	Role Emotional (RE)	Energy/ Fatigue (VT)	Emotional Well-being (MH)	Social Functioning (SF)	Pain (BP)	General Health (GH)	PCS	MCS	Health Change	Notes
Akowuah et al., 2023 [18]	Mitral valve repair: Minithoracotomy (n=166) vs Sternotomy (n=164); FU 6, 12, 18, 38 wk, 12 mo	EQ-5D	Baseline 35.29 → 1 y 45.67; Δ +10.12 (95% CI 7.90–12.37); p<0.001; between-arm Δ at 1 y: 0.10 (–2.59–2.81), p=0.94	Not reported	Not reported	Not reported	Not reported	Not reported	Not reported	Not reported	Not reported	Not reported	Not reported	SF-36 v.2 reports PF only as T scores; FU assessments with variable N; changes estimated via linear mixed-effects models
Purkayastha et al., 2021 [19]	Surgical AVR with Trifecta bioprosthesis; n=47; mean FU 3±1.7 years	no	68.42±21.54;	68.14±43.65	70.47±44.20;	68.42±16.07;	81.08±17.85;	68.21±18.50;	71.98±16.45;	59.14±19.73;	69.24±23.73	69.72±24.35	65.71±25.41	RAND SF-36 (0–100); Single postoperative assessment only (no pre-op baseline); composites reported as PHC/MHC mapped to PCS/MCS; HRQoL dataset available for 35 survivors (87.5% of initial dataset).
Kitamura et al., 2021 [20]	Edge-to-edge TTVR (MitraClip/TriClip/Pascal); n=115; FU 1 mo (subsets 6 mo n=72, 12 mo n=30)	MLHFQ	35.8 ±26.3 → 45.3 ±27.1; Δ +9.4 (4.4–14.4); p<0.001	17.2 ±31.0 → 35.9 ±41.0; Δ +18.7 (10.5–26.9); p<0.001	70.7 ±42.8 → 79.7 ±37.4; Δ +9.0 (0.3–17.7); p=0.044	36.9 ±19.1 → 38.2 ±20.5; Δ +1.3 (–2.8–5.3); p=0.53	68.2 ±17.4 → 71.0 ±17.4; Δ +2.8 (–0.6–6.1); p=0.10	81.3 ±23.1 → 86.5 ±21.4; Δ +5.3 (1.0–9.6); p=0.017	70.9 ±30.6 → 69.3 ±30.7; Δ –1.5 (–8.0–5.0); p=0.64	42.4 ±15.1 → 53.1 ±15.8; Δ +10.7 (7.1–14.3); p<0.001	33.7 ±8.9 → 36.7 ±9.0; Δ +3.0 (1.2–4.8); p=0.001	49.1 ±8.8 → 50.6 ±9.5; Δ +2.0 (0.4–3.7); p=0.017	Not reported	sample size decreases at 6/12 mo
Huang et al., 2020 [21]	Surgical Mechanical MVR: SJM (n=87) vs Star GK (n=85); FU 12 months	no	SJM 77.09±25.68 vs Star GK 75.76±24.05; p=0.55	SJM 70.78±20.15 vs Star GK 67.76±20.33; p=0.93	SJM 77.62±17.14 vs Star GK 76.15±16.89; p=0.90	SJM 60.56±20.21 vs Star GK 62.91±19.18; p=0.63	SJM 83.45±23.76 vs Star GK 79.51±22.45; p=0.27	SJM 88.14±24.24 vs Star GK 87.98±23.20; p=0.69	SJM 81.74±24.94 vs Star GK 77.36±25.61; p=0.81	SJM 66.20±19.21 vs Star GK 63.47±18.94; p=0.90	Not reported	Not reported	Not reported	Chinese SF-36 (0-100)
Wang et al., 2020 [22]	Surgical AVR: Biological (n=38) vs Mechanical (n=40); FU discharge & 1 year	CAQ questionnaire	Pre-op: Bio 63.5±12.1 vs Mech 64.3±11.3 (p=0.647). 1-y: Bio 83.4±15.4 vs Mech 70.3±11.3 (p=0.035). Δ: Not reported	Pre-op: 68.3±11.8 vs 68.4±15.2 (p=0.728). 1-y: 86.5±18.7 vs 76.5±13.6 (p=0.024). Δ: Not reported	Pre-op: 67.8±13.6 vs 66.5±17.8 (p=0.563). 1-y: 88.2±18.4 vs 76.6±15.9 (p=0.018). Δ: Not reported	Pre-op: 63.1±17.2 vs 62.5±14.1 (p=0.389). 1-y: 83.6±16.7 vs 72.7±14.2 (p=0.021). Δ: Not reported	Pre-op: 61.6±14.2 vs 60.4±12.6 (p=0.601). 1-y: 83.7±16.2 vs 71.6±14.5 (p=0.029). Δ: Not reported	Pre-op: 65.4±13.2 vs 67.3±16.6 (p=0.829). 1-y: 86.5±15.5 vs 75.2±17.3 (p=0.040). Δ: Not reported	Pre-op: 86.2±13.7 vs 84.6±10.6 (p=0.682). 1-y: 67.2±10.4 vs 66.8±12.4 (p=0.712). Δ: Not reported	Pre-op: 60.3±14.8 vs 61.2±13.8 (p=0.572). 1-y: 84.2±15.2 vs 73.6±15.5 (p=0.032). Δ: Not reported	Not reported	Not reported	Not reported	Chinese SF-36 (0-100). Authors state SF-36 was administered at discharge and at 1 year; however, Table 2 is labeled ‘pre-operative’—we interpret Table 2 as discharge per the Results text. Within-group change (Δ) was not reported.
Bento et al., 2019 [23]	Surgical AVR (sternotomy) in octogenarians with severe AS; n=81; assessments at baseline, 3, 6, 12 months	No	33.3 → 3 mo 59.6 (Δ +26.3; p<0.001) / 6 mo 59.7 (Δ +26.4; p<0.001) / 12 mo 62.0 (Δ +28.7; p<0.001)	31.7 → 3 mo 60.6 (Δ +28.9; p<0.001) / 6 mo 60.1 (Δ +28.4; p<0.001) / 12 mo 61.7 (Δ +30.0; p<0.001)	59.2 → 3 mo 76.6 (Δ +17.4; p<0.001) / 6 mo 74.0 (Δ +14.8; p<0.001) / 12 mo 73.9 (Δ +14.7; p=0.002)	35.3 → 3 mo 57.6 (Δ +22.3; p<0.001) / 6 mo 57.9 (Δ +22.6; p<0.001) / 12 mo 60.4 (Δ +25.1; p<0.001)	56.5 → 3 mo 64.6 (Δ +8.1; p=0.001) / 6 mo 66.1 (Δ +9.6; p<0.001) / 12 mo 63.4 (Δ +6.9; p=0.003)	67.4 → 3 mo 80.0 (Δ +12.6; p<0.001) / 6 mo 82.6 (Δ +15.2; p<0.001) / 12 mo 82.4 (Δ +15.0; p<0.001)	56.2 → 3 mo 81.2 (Δ +25.0; p<0.001) / 6 mo 80.8 (Δ +24.6; p<0.001) / 12 mo 80.7 (Δ +24.5; p<0.001)	47.6 → 3 mo 58.0 (Δ +10.4; p<0.001) / 6 mo 54.2 (Δ +6.6; p=0.016) / 12 mo 56.0 (Δ +8.4; p=0.001)	52.5 → 3 mo 55.3 (Δ +2.8; p<0.001) / 6 mo 55.0 (Δ +2.5; p<0.001) / 12 mo 55.3 (Δ +2.8; p<0.001)	56.1 → 3 mo 56.7 (Δ +0.6; p=0.342) / 6 mo 56.9 (Δ +0.8; p=0.011) / 12 mo 56.7 (Δ +0.6; p=0.076)	Not reported	Within-group change vs baseline at 3/6/12 months; all domains improved significantly (p≤0.003) except PCS
Huang LC et al., 2020 [24]	Mitral valve surgery: Totally endoscopic (EA, n=78) vs Median sternotomy (SA, n=85); 3 months	NRS and the SCAR scale	77.75 ± 8.08 vs 77.46 ± 8.08; between-group p=0.82	71.15 ± 15.12 vs 68.82 ± 15.39; p=0.33	65.14 ± 17.86 vs 62.18 ± 13.97; p=0.24	64.17 ± 11.99 vs 63.29 ± 11.51; p=0.64	74.62 ± 13.63 vs 68.42 ± 17.95; p=0.015	71.71 ± 12.20 vs 70.38 ± 11.87; p=0.48	77.05 ± 14.78 vs 70.12 ± 12.58; p=0.001	65.13 ± 13.31 vs 63.29 ± 13.51; p=0.347	Not reported	Not reported	Not reported	Chinese SF-36 (0-100); 3-month assessment and between-group comparisons only;
Korteland et al., 2016 [25]	Aortic valve replacement; non-elderly adults (18–60 at surgery); cross-sectional 1–10 years post-AVR; Mechanical (n=190) vs Biological (n=50)	Valve-specific questionnaire	49 ±10 vs 49 ±11	47 ±15 vs 43 ±16	49 ±14 vs 48 ±15	55 ±10 vs 53 ±10	53 ±10 vs 52 ±10	51 ±9 vs 48 ±11	54 ±9 vs 54 ±10	48 ±11 vs 44 ±12; p<0.05	48 ±9 vs 46 ±12	53 ±10 vs 52 ±10	Not reported	Dutch SF-36 (0-100); between-group differences largely non-significant—only GH was higher with mechanical valves (p<0.05). No baseline or paired Δ available.
Blokzijl et al., 2021 [26]	Elective SAVR, multicenter NL; baseline & ~1-year QOL; subscales stratified by age (<65 n=232; 65–79 n=554; ≥80 n=113)	no	<65: 57.6→81.6; Δ +24.0; p<.001. p	<65: 29.0→45.0; Δ +16.0; p<.001. p	<65: 44.0→50.5; Δ +6.5; p<.001. p	<65: 53.5→63.3; Δ +9.8; p<.001. p	<65: 62.9→73.0; Δ +10.1; p<.001. p	<65: 73.0→84.1; Δ +11.1; p<.001. p	<65: 75.5→87.4; Δ +11.9; p<.001. p	<65: 65.1→73.2; Δ +8.1; p<.001. p	Not reported	Not reported	Not reported	SF-36v2 (0-100); Δ values are derived from reported means.
Baron et al., 2019 [27]	TAVR (n=494) vs SAVR (n=449); assessments at baseline, 1, 6, 12 months	EQ-5D and KCCQ	Not reported	Not reported	Not reported	Not reported	Not reported	Not reported	Not reported	Not reported	TAVR: Δ +5.0 (1 m), +5.9 (6 m), +5.2 (12 m); all p<0.001 vs BL. SAVR: Δ –2.7 (1 m; p<0.001 decrease), +5.1 (6 m; p<0.001), +5.0 (12 m; p<0.001).	TAVR: Δ +3.4 (1 m; p<0.001), +3.5 (6 m; p<0.001), +3.5 (12 m; p<0.001). SAVR: Δ +0.1 (1 m; p=0.921 ns), +4.5 (6 m; p<0.001), +4.0 (12 m; p<0.001).	Not reported	SF-36 detailed domain not reported, baseline also not reported only Δ —PCS/MCS only (paired Δ).
Abd Al Jawad et al., 2022 [28]	AVR: ministernotomy (n=96) vs minithoracotomy (n=93), FU 12 mo	no	70.3 ±22.9 → 79.7 ±20.7, Δ +9.4, p=0.0036	58.6 ±31.2 → 75.3 ±26.5, Δ +16.7, p=0.0001	78.8 ±26.1 → 82.3 ±24.2, Δ +3.5, p=0.3366	66.9 ±19.9 → 66.0 ±10.5, Δ -0.9, p=0.6965	78.3 ±17.2 → 74.8 ±17.1, Δ -3.5, p=0.1617	66.2 ±27.3 → 87.9 ±17.0, Δ +21.8, p<0.0001	78.1 ±21.4 → 83.5 ±17.4, Δ +5.4, p=0.0611	66.3 ±22.2 → 61.0 ±14.2, Δ -5.3, p=0.0532	Not reported	Nor reported	83.6 ±22.6 → 93.3 ±11.7, Δ +9.7, p=0.0003	Arabic SF-36 (0-100)
Sævik et al., 2025 [29]	Transfemoral TAVI; n=88; FU 12 mo	no	54.2 (49.4–59.0) → 70.4 (66.0–74.8);	48.5 (42.4–54.6) → 62.6 (56.1–69.1);	71.7 (65.5–77.8) → 80.2 (74.4–86.1);	44.0 (38.9–49.0) → 54.5 (49.3–59.6);	78.2 (74.6–81.8) → 80.3 (76.0–84.6);	71.5 (66.19-76.99)	63.95 (58.14-69.76) → 70.44 (65.04-75.83) Δ + 6.90 (1.49-12.30); p=0.120	62.06 (57.94-66.18)	40.97 (39.02-42.93)	50.19 (48.07-52.30)	Not reported	Norwegian SF-36 v2 (0-100); 12-mo paired Δ with 95% CI

In addition to data extraction, we qualitatively assessed the risk of bias in included studies. This was based on study design (prospective vs. retrospective), sample size, completeness of reporting, and clarity of outcome measurement. No formal risk-of-bias tool was applied, but potential sources of bias were considered in the interpretation of findings.

Studies were categorized as having ‘Low,’ ‘Moderate,’ or ‘High’ risk of bias based on the following operational criteria: Low risk: prospective design, ≥100 participants, complete SF-36 reporting at ≥2 time points, and clear description of inclusion criteria and outcomes; Moderate risk: prospective or retrospective design, 50-99 participants, minor missing data or reporting gaps; High risk: small sample size (<50 participants), retrospective analysis, substantial missing data, or unclear inclusion criteria.

Bias appraisal was independently performed by the first author and cross-checked by the senior author; disagreements were resolved by consensus. No inter-rater reliability tools (e.g., Cohen’s kappa) were used, given the qualitative nature of the review. Since no formal meta-analysis was performed, studies with a higher risk of bias were not excluded but were qualitatively downweighted in the narrative synthesis.

We did not perform funnel plot analysis or similar techniques for publication bias, but acknowledge that restricting to English-language, free full-text articles limits certainty and may favor positive findings. Missing or incomplete data in included studies did not lead to exclusion, but were noted and considered when interpreting the strength of evidence. Due to the heterogeneity of study methods, patients, and SF-36 reporting, we did not calculate certainty of evidence for each intervention using tools such as Grading of Recommendations Assessment, Development and Evaluation (GRADE), and thus these conclusions remain hypothesis-generating. This can be further seen in Table 4. 

**Table 4:** Qualitative appraisal AVR: aortic valve replacement; HRQOL: health-related quality of life; MR: mitral regurgitation; MVR: mitral valve replacement; QoL: quality of life; RCT: randomized controlled trial; SAVR: surgical aortic valve replacement; TAVR: transcatheter aortic valve replacement; TAVI: transcatheter aortic valve implantation; TMVR: transcatheter mitral valve replacement; TR: tricuspid regurgitation; TTVR: transcatheter tricuspid valve repair/replacement. Qualitative appraisal levels were as follows: High: strong methodological design (e.g., multicenter RCT or large prospective cohort); Moderate: acceptable design with some limitations (e.g., single-center, limited adjustment); Low: exploratory or retrospective studies with reduced generalizability.

Author, Year of publication	Key Limitations	Qualitative Appraisal
Akowuah et al., 2023 [18]	Open-label randomized surgical trial; expertise/learning-curve variation across centers; QoL primary at 12 weeks (short-term); degenerative MR population limits generalizability to valve replacement.	High; multicenter RCT with prespecified QoL endpoint and robust follow-up; limitations relate mainly to short-term QoL horizon and procedure expertise effects.
Purkayastha et al., 2021 [19]	Prospective single-center cohort without randomized comparator; modest sample with incomplete HRQoL at all timepoints; limited adjustment for confounding.	Moderate; prospective design and mid-term horizon, but single-center, no control, and limited adjustment.
Kitamura et al., 2021 [20]	Single-center, nonrandomized cohort; niche population (functional TR after TTVR); partial missingness at later timepoints; no control group.	Low–moderate; valuable exploratory data but limited by design, sample size, and generalizability.
Huang et al., 2020 [21]	Retrospective single-center comparison of two mechanical valves; no randomization; potential selection and reporting biases; limited external validity.	Moderate; clear SF-36 reporting and predefined timepoints, but retrospective design and confounding risks.
Wang et al., 2020 [22]	Retrospective single-center with small N; nonrandomized prosthesis selection; limited covariate adjustment; anxiety outcomes only at 1 year.	Low–moderate; informative age-specific cohort but constrained by design and limited adjustment.
Bento et al., 2019 [23]	Single-center retrospective study; octogenarian cohort limits generalizability; small-to-moderate sample; no contemporaneous TAVR/SAVR comparisons.	Moderate; consistent serial follow-up, but methodological limitations and a narrow population.
Huang LC et al., 2020 [24]	Retrospective single-center with procedure-selection bias; short follow-up; limited adjustment for baseline differences.	Low–moderate; clear SF-36 reporting, but short horizon and selection bias.
Korteland et al., 2016 [25]	Cross-sectional postal survey; no preoperative baseline; survivor/response bias; limited causal inference.	Moderate; sizable cohort and long post-AVR window, but design precludes longitudinal change estimation.
Blokzijl et al., 2021 [26]	Observational multicenter cohort; elective cases only; residual confounding across age strata; limited beyond-one-year data.	Moderate; good size and multicenter design with baseline/one-year pairing, but nonrandomized and one-year horizon.
Baron et al., 2019 [27]	Industry involvement; unblinded treatment allocation; low-risk cohort limits applicability to higher-risk patients.	High; large multicenter RCT with rigorous serial HRQoL assessments; minor concerns about generalizability.
Abd Al Jawad et al., 2022 [28]	Prospective but nonrandomized; questionnaire-based; comparison across minimally invasive incisions only; single-country setting.	Moderate; pragmatic, prospectively assembled cohort with one-year HRQOL, limited by design and scope.
Sævik et al., 2025 [29]	Single-center elderly cohort; no control group; loss to follow-up; exploratory threshold for clinically meaningful improvement.	Moderate–High – prospective design with predefined HRQOL domains; some attrition and limited external validity.

The research results across various studies highlight the impact of different surgical approaches and valve replacement techniques on patient outcomes, specifically in terms of physical and mental health as measured by the SF-36 questionnaire.

Akowuah et al. (2023) [18] reported physical functioning T-scores only; both mini-thoracotomy and sternotomy for mitral valve repair showed improvement in physical function scores up to one year, with similar efficacy between the two interventions. Purkayastha et al. (2021) [19] found that patients undergoing aortic valve replacement with the Trifecta bioprosthetic valve showed good mental and physical health at an average follow-up of three years. Kitamura et al. (2021) [20] observed the largest improvements in physical role and general health scores among patients undergoing transcatheter tricuspid valve repair, indicating effective addressal of physical limitations and overall well-being.

Huang et al. (2020) [21] noted good mental and physical health outcomes among patients after mechanical mitral valve replacement, with no significant differences between the St. Jude Medical (SJM) and Star GK valves. Wang et al. (2020) [22] revealed that patients with biological valve replacements exhibited better long-term quality of life and lower levels of cardiac anxiety compared to those with mechanical valves. Bento et al. (2019) [23] showed significant improvements in all dimensions of SF-36 among octogenarians undergoing aortic valve replacement, with notable reductions in mental burden post-surgery.

Huang et al. (2020) [24] found significant differences in bodily pain and mental health subscales favoring the modified total endoscopic approach over median sternotomy for mitral valve repair. Korteland et al. (2016) [25] reported that aortic valve replacement patients had lower physical but higher mental health scores compared to the general population, with an association between increased time post-surgery and improved mental health. Blokzijl et al. (2021) [26] highlighted improvements in both physical and mental health scores after surgery across all patient age groups, with older patients experiencing a decline in physical health at one year.

Baron et al. (2019) [27] indicated significant early post-procedure improvements in physical and mental health for transcatheter over surgical patients, but with comparable long-term outcomes between the two groups. Abd Al Jawad et al. (2022) [28] demonstrated significant health improvements in the mini-thoracotomy group compared to the mini-sternotomy group, suggesting superior physical health outcomes with the mini-thoracotomy approach.

Sævik et al. (2025) [29] investigated 88 elderly patients with severe aortic stenosis undergoing transfemoral TAVI. Using the SF-36 questionnaire at baseline and 12 months, they reported significant improvements in physical functioning and role limitations due to physical problems, with approximately half of the cohort achieving clinically meaningful improvement (≥15 points). Additional benefits were observed in general health, vitality, social functioning, and the physical component score, while baseline impairments predicted greater postoperative gains.

Overall, the majority of included studies were prospective observational cohorts with moderate sample sizes. Retrospective analyses and studies with limited follow-up were considered at higher risk of bias. These findings underscore the importance of considering both the type of surgical intervention and the patient's specific health profile when planning cardiac surgeries. They also highlight the potential benefits of minimally invasive procedures and the need for comprehensive postoperative care to enhance patient recovery and quality of life.

Analyzing the research results presented, some common trends, divergences, and specific additions can be observed between the studies, which provide a rich perspective on the care of cardiac patients.

Similarities

Most studies report an improvement in patients' physical and mental health after surgery, whether it is aortic valve replacement, mitral valve repair, or tricuspid valve repair, highlighting the positive impact of surgery on patients' quality of life.

Almost all studies used the SF-36 questionnaire to assess quality of life, indicating the wide acceptance of this instrument as a standard in assessing the well-being of patients after cardiac interventions.

Divergences

Some studies, such as Akowuah et al. (2023) [18] and Abd Al Jawad et al. (2022) [28], directly compare the effectiveness and impact of different surgical approaches (mini-thoracotomy vs. sternotomy, mini-thoracotomy vs. mini-sternotomy) on patient recovery, while other studies focus on the results of a single method. This variety reflects the diversity in surgical research and the need to best understand the approach for each patient.

Wang et al. (2020) [22] and Korteland et al. (2016) [25] explore the differences between the use of mechanical and biological valves, showing different outcomes in terms of cardiac anxiety and long-term quality of life. These differences emphasize the importance of valve type selection based on individual patient needs.

Additions

In addition to the SF-36, some studies integrate additional assessment tools, such as the Minnesota Living with Heart Failure Questionnaire (MLHFQ; Kitamura et al., 2021) [20] or EQ-5D (Baron et al., 2019) [27], providing a more complex picture of the impact of interventions on patients' quality of life.

Blokzijl et al. (2021) [26] discuss the influence of age on long-term outcomes, an important dimension that can guide treatment strategies for different age groups.

These studies collectively emphasize the importance of choosing the surgical method and valve type according to the specific patient profile to optimize postoperative outcomes and quality of life. They also highlight the value of multidimensional assessment of patients' well-being, using both standardized and specific instruments to capture a wide range of effects of surgical interventions. Ultimately, tailoring the surgical approach and postoperative care to the individual needs of each patient appears to be the key to maximizing the long-term benefits of cardiac treatment.

Discussion

Across the 12 included studies, a consistent pattern emerged: irrespective of intervention type, patients demonstrated early postoperative improvements in SF-36 physical domains (typically 10-25 point increases), with mid-term stabilization and smaller gains in mental health scores. Studies directly comparing surgical and transcatheter approaches reported diminishing differences by six to 12 months, and subgroup analyses revealed attenuated improvements with increasing age. Given methodological and reporting heterogeneity, effect sizes were not pooled; instead, clinically meaningful changes were judged based on established SF-36 thresholds (≥10-point increases). Supplementary tools such as EQ-5D and KCCQ showed generally concordant trends in physical recovery, although these instruments emphasize disease-specific dimensions rather than global health.

Early HRQOL gains were consistent across interventions, with the largest and most reproducible improvements in physical functioning (≈10-25-point increase in the first 12 months) and moderate and variable gains in vitality and general health. By valve position, evidence is strongest for aortic interventions (surgical and TAVI), while mitral and especially tricuspid data remain more limited and heterogeneous; therefore, conclusions are more data-driven for aortic valve populations. Differences by procedural risk category were insufficiently and inconsistently reported; where baseline impairment/frailty was greater, absolute physical gains tended to be larger, but between-group adjusted comparisons by risk were rarely available. Mortality and rehospitalization were not uniformly reported alongside SF-36; when available, worse hard outcomes coincided with attenuated HRQOL gains, suggesting the value of joint reporting.

Comparing different surgical approaches for mitral valve repair, both mini-thoracotomy and sternotomy methods demonstrated significant improvements in physical function and quality of life over time. Additionally, endoscopic approaches showed potential advantages, including reduced postoperative pain and better mental health outcomes compared to median sternotomy [24].

With regard to aortic valve replacement procedures, whether surgical or transcatheter, all studies indicated substantial and sustained enhancements in quality of life across various patient demographics [19,20,22-28]. Across studies with stratified reporting, all age groups improved after intervention; however, absolute gains in the physical domains were attenuated with increasing age, and older groups remained at lower levels at follow-up (for example, in a multicenter surgical cohort, physical functioning Δ +24.0 in patients <65 years versus +14.5 in those ≥80 years; role physical Δ +16.0 versus +9.1; general health Δ +8.1 versus +5.2 at approximately one year) [26]. Changes in mental health were more modest and often similar across ages (for example, mental health Δ +10.1 in patients <65 years versus +2.5 in those ≥80 years at approximately one year) [26]. However, older patients demonstrated improvements in mental health scores over time, comparable to or exceeding those of younger patients [26]. Moreover, variations were observed in the choice of valve type and its impact on long-term outcomes, with younger patients and those undergoing specific repair procedures experiencing better scores [19,22].

Longer-term patterns beyond 12 months remain uncertain; cross-sectional surveys one to 10 years after aortic valve replacement suggest lower general health in biological versus mechanical valve recipients, but these comparisons are confounded by age and selection and do not establish trajectories [25].

Observational comparisons between mechanical and bioprosthetic valves are prone to confounding by indication: patients receiving bioprostheses are typically older and more comorbid, which independently depresses physical domains, whereas treatment-related factors in mechanical valves (lifelong anticoagulation, valve noise/audibility) may influence mental and pain domains [19,22,25]. Accordingly, observed differences at one year should not be attributed solely to prosthesis type. Future analyses should adjust for age, comorbidity burden, and anticoagulation (e.g., multivariable or propensity-based models) to better isolate valve-type effects.

Comparisons between transcatheter interventions and traditional surgical procedures showed comparable improvements in overall health status over time [20,26,27]. Notably, long-term follow-up revealed no significant differences in health status measures between transcatheter and surgical groups [28].

More recently, Sævik et al. [29] proposed a novel threshold for clinically meaningful improvement in the SF-36 physical domains following TAVI, reporting that approximately half of patients achieved significant long-term gains in physical functioning and role limitations due to physical problems. Importantly, those with the lowest baseline scores derived the greatest postoperative benefit, underscoring the value of preoperative HRQOL assessment in patient selection and counseling.

Other Patient-Reported Instruments

Several studies also reported non-SF-36 patient-reported measures. The EQ-5D provides a preference-based index (0-1) and a 0-100 Visual Analogue Scale; in a randomized low-risk aortic stenosis cohort, it showed an early advantage at one month for the transcatheter approach, with no consistent differences at six to twelve months [27]. The KCCQ is a disease-specific health status instrument (0-100 overall summary; higher is better) and captured larger early improvements and higher “excellent outcome” rates after transcatheter therapy in the same trial. [27] The CAQ assesses fear, avoidance, and heart-focused attention; at one year, higher anxiety scores were observed in mechanical valve recipients compared with bioprosthesis recipients [22]. The Numeric Rating Scale (NRS) for pain (0-10) and a scar assessment scale were used after totally endoscopic mitral surgery and favored the endoscopic approach. (24) For consistency, we tabulated only SF-36 outcomes, while these additional measures are summarized narratively and not pooled [22,24,27].

Implications and Future Directions

Future work should adopt standardized assessment windows (baseline, one, six, 12, and ≥24 months), report thresholds for clinically meaningful change at the domain level, integrate objective functional measures (for example, six-minute walk distance) with patient-reported outcomes to contextualize physical gains, and delineate longer-term mental-health trajectories beyond one year.

Study Limitations

The search strategy was restricted to English-language publications and free full texts. This may have led to the omission of relevant evidence and introduced publication and language bias. Also, no formal risk-of-bias or certainty-of-evidence tool, such as Risk Of Bias In Non-randomised Studies of Interventions (ROBINS-I) or GRADE, was applied. Instead, a qualitative appraisal was performed, noting that variability in study design, sample size, and reporting completeness may have introduced bias and should be considered when interpreting findings. The range of study designs, from retrospective to prospective, poses challenges in standardizing methodologies and patient characteristics, potentially hindering direct comparisons and generalizability. Limited sample sizes in some studies may compromise statistical power and applicability, leading to imprecise estimates of treatment effects and increased risk of type II errors. Variability in follow-up duration across studies complicates the assessment of intervention effectiveness and sustainability, with short-term versus long-term observations yielding diverse outcomes. Also, incomplete reporting of patient demographics and health status in certain studies limits the ability to adjust for potential confounders and interpret findings accurately. While the SF-36 questionnaire is widely used for assessing quality of life, variations in administration and interpretation across studies may introduce measurement biases. Furthermore, a formal assessment of publication bias (e.g., funnel plots) and small-study effects cannot be excluded. In addition, a quantitative meta-analysis was not conducted, given the substantial heterogeneity in study designs, populations, follow-up windows, and outcome reporting. Moreover, although some included studies reported outcomes from additional HRQOL instruments such as EQ-5D or KCCQ, our synthesis was restricted to SF-36 results, which may limit comparability across measures. Despite attempts to control for confounding variables, residual confounding remains a concern, potentially influencing study outcomes. Potential publication bias/underreporting of negative HRQOL results cannot be excluded, given the English-language, free full-text restriction and the narrative design. To refine estimates and enable adjusted comparisons, future research should prioritize multicenter prospective designs, standardized follow-up windows (baseline, one, six, 12, ≥24 months), uniform SF-36 reporting at the domain level, and adequate sample sizes per stratum (e.g., ≥100 per valve/approach). Patient-level pooled analyses (IPD) or harmonized meta-analytic approaches are warranted to address confounding (age, frailty, anticoagulation) and to test subgroup effects (valve type/position, surgical vs. transcatheter). Finally, embedding HRQOL endpoints in cost-effectiveness and policy frameworks could strengthen guideline relevance and resource allocation.

## Conclusions

Following this review, several consistent patterns emerge. Early (≤1 month) improvements in patient-reported health status are evident after both surgical and transcatheter heart valve interventions, with the greatest gains typically seen in the physical domains of the SF-36, as shown in randomized and prospective cohorts. Mid-term (six to 12 months) improvements are generally sustained, and differences between transcatheter and surgical approaches tend to diminish over time, with no consistent between-group differences by one year in studies reporting adjusted analyses. Longer-term (>12 months) trajectories remain less certain, as available data are limited, often cross-sectional, and potentially confounded by age and selection bias.

Overall, the evidence base is constrained by heterogeneity in study design, populations, follow-up intervals, and outcome reporting, as well as the absence of formal risk-of-bias assessment. These conclusions are therefore conservative and should be viewed as hypothesis-generating. Future research should adopt standardized patient-reported outcome time points (baseline, one, six, 12, and ≥24 months) and prespecified thresholds for clinically meaningful change to strengthen comparability and guide shared decision-making. Clinically, optimizing patient benefit requires aligning intervention choice with age, comorbidity burden, and individual priorities, complemented by structured rehabilitation and mental health support.
